# Efficacy of Melatonin as a Sleep Inducer in EEG Procedures in the Pediatric Population: A Cross-Sectional Study

**DOI:** 10.7759/cureus.54196

**Published:** 2024-02-14

**Authors:** Saja Tahir, Kate Flynn, Mohamed O E Babiker

**Affiliations:** 1 Department of Pediatrics, Al Jalila Children's Speciality Hospital, Dubai, ARE; 2 Department of Neurology, Al Jalila Children's Speciality Hospital, Dubai, ARE

**Keywords:** neurology, sleep eeg, pediatric neurology, electroencephalography (eeg), melatonin

## Abstract

Introduction

Melatonin has been used as an alternative to sleep deprivation for EEG sleep induction in the pediatric population. Our study aims to describe the efficacy of the currently used doses of melatonin for sleep induction among the pediatric age group.

Methods

A retrospective cross-sectional study included all patients who underwent an EEG after receiving melatonin over the period of one year. A total of 126 patients have been included in the study. Patients aged one year to three years received oral melatonin in doses between 2 mg and 6 mg. Patients in the age of three years and above received 10 mg of melatonin. Patients’ success rate in achieving sleep and the exact time required for the patients to fall asleep were obtained using the readings of their EEG. The percentage of patients who have achieved sleep and the time required for those patients to sleep were calculated and correlated with the patient's gender, the presence of any associated neurobehavioral disorders, and their use of antiepileptic drugs (AED).

Results

Successful sleep was achieved in 84.9% (n:107) of the patients, with a mean time of 24 minutes to fall asleep (SD =* *14.36). Patients with neurobehavioral disorders were 20% less likely to fall asleep when compared to other patients without neurobehavioral disorders (p: 0.003). However, there was not a statistically significant difference among different genders and among patients who received AED.

Conclusion

Melatonin is an effective sleep inducer for patients undergoing EEG procedures. It should be considered in the majority of patients. However, in patients with neurobehavioral disorders, a lower success rate is expected.

## Introduction

Sleep EEG is a valuable assessment for the diagnosis of multiple neurological conditions in children. In fact, some conditions can only be diagnosed during a sleep EEG. In addition, sleep EEG minimizes the presence of artifacts due to the relative lack of cooperation in young age groups. However, achieving sleep imposes a significant challenge in the pediatric population. Sleep is usually achieved either by administration of sleep-inducing medications or by partial sleep deprivation. In many instances, the latter cannot be achieved or is perceived as extremely burdensome by the family [1]. Pharmacological sedation cannot be used as the majority of the medications tend to affect the brain's electrical activity thus the interpretation of the EEG records. Melatonin on the other hand is a safe sleep inducer that has been successfully used in EEG sleep induction and was found to be as effective as sleep deprivation [1]. The aim of this study is to describe the efficacy of melatonin as a sleep inducer among the pediatric age groups of children undergoing EEG procedures. Of note, this article was previously presented as a poster presentation at the British Pediatric Neurology Association (BPNA) Annual Conference on 25 January 2023 in Edinburgh, United Kingdom. 

## Materials and methods

Study design

A retrospective cross-sectional study was conducted using the records of all the patients who received melatonin prior to their EEG procedures in Al Jalila Children’s Hospital during the year 2021. A total of 126 patients were included in the study.

Patients were not requested to be sleep-deprived prior to starting the procedure. All patients received their melatonin dose within 30 minutes of starting the EEG procedure. Patients above the age of three years received melatonin in the dose of 10 mg, while patients in the age group of one year to three years received melatonin in the doses of 2 mg, 3 mg, 4 mg, 5 mg, and 6 mg. Infants below one year of age were left to naturally sleep without melatonin administration.

The success of the patients to achieve sleep and the time required for the patients to achieve sleep were obtained by calculating the time required for patients to record EEG sleep patterns such as sleep spindles, vertex sharp waves and K-complexes. The number of patients who fell asleep, and the time needed for those patients to fall asleep were both calculated and correlated to patients’ gender, neurobehavioral disorders, and the use of antiepileptic drugs (AED).

Study participants

This sample included all patients in the age group between one year to 18 years who underwent an EEG after receiving a single dose of melatonin for the duration of one year. Patients who had chloral hydrate instead of melatonin and patients with missing data were excluded from the study.

Statistical analysis

Data analysis was conducted using SPSS Statistics version 27 (IBM Corp., Armonk, NY). The demographic characteristics of the participants were described using frequency and percentage. The duration of sleep was described using means and standard deviations (SDs).

To assess the relationship between sleep success percentage and gender, AED usage, and the presence of neurobehavioral disorders, chi-square tests were employed to compare different groups. Pearson correlation analysis was utilized to determine the correlation between the mean time needed to achieve sleep and variables including gender, AED usage, and neurobehavioral disorders. Statistical significance was set at a p-value of less than 0.05.

## Results

We had a total of 126 children and adolescents undergoing EEG procedures. Out of them, 107 patients (84.9%) have successfully slept while 19 patients (15%) failed to achieve sleep. In terms of the required time to fall asleep, 24 minutes was the mean time (SD =* *14.36) required for those patients to sleep.

The study included an equal number of males and females with no significant difference in achieving sleep between the two groups. Sixty-three patients (50%) were females, and 63 patients (50%) were males (Table 1). 79.4% (n:50) of males slept, while 90% (n:57) of females slept. Whether the patient was a male or a female, the mean time required to sleep had no significant difference. The mean time required to sleep for males was 24.82 minutes (SD = 12.8 ) and 23.32 minutes for females (SD = 15.3) (Table 2).

**Table 1:** Demographic data of the participants

Variable	No.	Percentage %
Gender		
Male	63	50
Female	63	50
Diagnosis		
With neurobehavioral disorders	42	33.3
Without neurobehavioral disorders	84	66.7
Medications		
With antiepileptics	54	42.9
Without antiepileptics	72	57.1

**Table 2:** Relationship between patient variables with the sleep success percentage and the mean time needed to sleep SD, standard deviation

Variable	Sleep success %	P-value	Mean time to sleep in minutes	SD
Gender				
Male	79.4	0.081	24.82 minutes	12.8
Female	90.5		23.32 minutes	15.3
Diagnosis				
With neurobehavioral disorders	71.4	0.003	19.87 minutes	14.6
Without neurobehavioral disorders	91.7		25.64 minutes	19.8
Medications				
With antiepileptics	85.2	0.943	22.5 minutes	14.6
Without antiepileptics	84.7		25.16 minutes	14.16

In terms of the use of AEDs, our sample included 54 patients (42.9%) who were receiving AEDs (Table 1). Out of those, 46 (85.2%) patients fell asleep with a mean time of 22 minutes (SD = 14.6), showing no difference from patients who were not receiving AEDs (M = 25.16 minutes, SD =* *14.16) (Table 2).

Our sample included 42 patients (33.3%) with neurobehavioral disorders (Table 1). Patients with neurobehavioral disorders were less likely to sleep with a 20.3% statistically significant difference (p: 0.003) (Table 2). However, when analyzing the time required to sleep, they required a shorter mean time of 19.8 minutes (SD* *= 14.6), compared to 25.6 minutes in patients without neurobehavioral disorders (SD* *= 19.8) (Table 2). Although this is a statistically significant difference (p: 0.115), this finding might indicate that patients with neurobehavioral disorders have difficulty initiating sleep not in the time required to fall asleep. We suggest that this significance might be proven with a larger sample size.

## Discussion

Sleep-related changes are widely recognized as a significant trigger for seizures. These changes pertaining to sleep can influence various aspects of seizures, including their initiation, frequency patterns, seizure types, and the observed changes in the EEG readings [2].

The occurrence of epileptiform abnormalities in a standard initial EEG is found to range from 20% to 50% among individuals suspected of having epilepsy, regardless of their age [3]. To enhance the diagnostic effectiveness of routine EEG, it is recommended to perform repetitive EEG recordings and employ activation techniques. Research suggests that the likelihood of detecting epileptiform activity rises to around 80% when EEG is conducted during sleep [4]. It is worth noting that certain forms of epilepsy, such as Childhood Epilepsy with Centrotemporal Spikes (CECTS) or Landau-Kleffner syndrome, may exclusively exhibit abnormalities during sleep.

In order to successfully detect these EEG findings in children, sleep is usually induced by the conventional methods of partial sleep deprivation or by the administration of a sleep-inducing agent. Various medications, such as chloral hydrate, clonidine, barbiturates, and chlorpromazine, have been utilized to induce sleep. However, it is important to note that many of these pharmacological agents can potentially have adverse effects and can influence the overall structure of sleep, which may have an impact on the analysis of the EEG data obtained during sleep recordings [5].

It is important to acknowledge that many of these sleep-inducing medications have potential side effects and varying rates of success. One such medication is chloral hydrate, which is frequently employed as a sleep-inducing agent. The reported rates of success in inducing sleep with chloral hydrate have been inconsistent. For instance, Napoli et al. found an 84% success rate when administering chloral hydrate to pediatric patients undergoing cardiac echocardiography. Conversely, Weir et al. achieved an 80% success rate in inducing sleep with chloral hydrate among the pediatric population as a sedative. Additionally, Rumm et al. reported a success rate of 73% when using chloral hydrate in pediatric patients with neurological disorders [6-8].

Regarding the possible side effects of chloral hydrate, it is worth noting that in a minority of children, respiratory depression, hypoxemia, and possible apnea have been reported with the use of chloral hydrate.

Reports indicate that the incidence of respiratory depression associated with chloral hydrate can reach up to 4% [9,10]. 

Melatonin offers a safe alternative for sleep induction instead of the currently used methods. Melatonin is an endogenous hormone produced in the pineal gland that is secreted in the dark to help regulate the body’s natural sleep/wake cycle [11]. An exogenous form of the drug has been used to induce natural sleep for different procedures including EEG procedures and magnetic resonance imaging (MRI) [12].

Our study demonstrates that melatonin in doses between 2 mg to 6 mg in the age group between one to three years and in the dose of 10 mg in the age group above three years is an effective sleep inducer for EEG procedures. It resulted in successful sleep in 84.9% of our patients. This result is consistent with a study reported by Wassmer et al. (2000), which described that melatonin may be used in children to induce sleep during EEG recordings effectively [11].

The mean time to achieve successful sleep in our study was 24 minutes. In a similar study, which was conducted to report the efficacy of melatonin as a sleep inducer for MRI procedures, patients slept within 35 minutes. However, this study was conducted on a smaller sample size [12].

The presence of a neurobehavioral disorder was the main factor affecting the success rate of patients to sleep. Patients with neurobehavioral disorders were 20% less likely to fall asleep when compared to other patients without neurobehavioral disorders. These disorders included intellectual disability, global developmental delay, attention deficit hyperactivity disorder (ADHD), autism spectrum disorder (ASD), anxiety, and psychosis. These findings were previously reported by Baddam et al. (2018), who reported high sleep onset latency, short sleep duration, and resistance to bedtime in patients with anxiety, ADHD, and ASD [13]. Therefore, we suggest considering an alternative sleep inducer in this cohort of patients.

On the other hand, our results suggest that the use of AED should not prevent or affect the use of melatonin as a sleep inducer. In our sample, there was no difference in the success rate of achieving sleep nor the time required to sleep between patients who received AEDs and the patients who did not.

There are several limitations to consider in our retrospective study investigating the use of melatonin as a sleep inducer for EEG procedures. First, the inclusion of a wide range of age groups makes it difficult to study the heterogeneous response to melatonin among different ages, as sleep onset and sleep patterns can vary across different developmental stages [14]. Second, the inclusion of a wide range of melatonin dosing regimens further impedes the exact analysis of the specific dose effect on sleep induction. Finally, due to the retrospective nature of the study, we were unable to obtain precise information on the time needed for participants to achieve full wakefulness after melatonin administration. These limitations should be considered when interpreting the findings of our study.

## Conclusions

In conclusion, our study provides strong evidence supporting the effectiveness of melatonin as a sleep inducer for patients undergoing EEG procedures. The data clearly demonstrates a remarkable success rate of 84.9% in inducing sleep among the pediatric population when administered within 30 minutes of initiating the EEG, even without the need for pre-procedure sleep deprivation. This data supports that melatonin administration is a reliable and convenient method for sleep induction in most patients. However, it is important to note that our findings indicate a lower success rate in patients with neurobehavioral disorders. Therefore, in such cases, the consideration of additional or alternative sleep-inducing methods is warranted to ensure optimal results. Overall, our study highlights the valuable role of melatonin in facilitating sleep for EEG procedures, but individual patient characteristics should be carefully considered for tailored approaches to maximize its effectiveness.
